# Change in influenza vaccine uptake among adults in the United States from May 2020 to October 2024

**DOI:** 10.1371/journal.pgph.0004756

**Published:** 2025-07-16

**Authors:** Hannah Melchinger, Sameer M. Belgaumi, Noureen Ahmed, Saad B. Omer, Amyn A. Malik

**Affiliations:** Peter O’Donnell Jr. School of Public Health, UT Southwestern Medical Center, Dallas, Texas, United States of America; University of Michigan, UNITED STATES OF AMERICA

## Abstract

Influenza vaccines are essential against the spread of influenza virus. In the United States, the Centers for Disease Control and Prevention (CDC) recommends vaccination to all eligible individuals over 6 months of age; however, uptake remains suboptimal nationally. We do not fully understand the current nuances of influenza vaccine uptake, nor how it has been affected by the coronavirus disease 2019 (COVID-19) pandemic. From 2020-2024, we conducted two distinct, cross-sectional surveys assessing influenza vaccine uptake among US adults. A census-matched national sample was recruited by online survey company CloudResearch, and respondents were asked whether they had or planned to receive the influenza vaccine. Analyses were stratified by demographic and weighted according to age, sex, and race estimates from the American Community Survey (ACS) 2022. We found that overall uptake decreased, with significant declines among demographic groups usually associated with higher vaccine uptake. Compared to 2022, individuals who were older (11% decrease), male (13%), White (7%), non-Hispanic (5%), or more educated (16%) were significantly less likely to receive the influenza vaccine in 2024. Changes in attitudes and intentions towards vaccination during and since the COVID-19 pandemic have been associated with several factors, including changes in perceived risk and the rise of vaccine-related mis- and disinformation. Targeted behavioral interventions can be used to shift attitudes, intentions, and eventually, behaviors, towards health-seeking behaviors like vaccination. We must target these demographics with evidence-based behavioral interventions to improve uptake of influenza vaccination.

## Introduction

Immunization is one of public health’s most effective tools against infectious diseases, preventing an estimated 3.5 to 5 million deaths due to infectious disease each year [1]. The influenza vaccine is a common annual vaccine against the influenza virus, a respiratory disease which, in severe cases, can result in hospitalization or death. Between 2010 and 2024, influenza is estimated to have caused 9.3 million – 41 million illnesses, 120,000 – 710,000 hospitalizations, and 6,300 – 52,000 deaths annually [2]. In the United States (US), the Centers for Disease Control and Prevention (CDC) recommends the influenza vaccine for all people over the age of 6 months who do not have contraindications [3].

Vaccination can reduce severe outcomes associated with influenza infection, especially among the elderly [4]. During the 2023 – 2024 season, the CDC estimates that influenza vaccination prevented 9.8 million related illnesses, 4.8 million medical visits, 120,000 hospitalizations, and 7,900 deaths [5]. However, influenza vaccine uptake among American adults is suboptimal. In 2023 – 2024, only about 45% of US adults over the age of 18 were vaccinated against influenza, two percentage points lower than in 2022 – 2023 [6] Historically, coverage has been higher among American adults who are female, White, or older [6–8]. These metrics fall significantly short of the Healthy People 2030 goal of 70% coverage nationally, highlighting an urgent need to improve uptake across the country [8–9].

As with many essential health services and immunization schedules, seasonal influenza immunizations were interrupted by the coronavirus disease 2019 (COVID-19) pandemic [10–11]. The direct impact of the COVID-19 pandemic on influenza vaccination status and intention among US adults is unclear: between 2020 and 2022, influenza vaccination rates among US adults dropped by three percentage points (50%–47%), notably among at-risk populations including pregnant women (55%–47%) and older adults (75%–70%) [12]. Coverage among healthcare professionals fluctuated between 2019 and 2023, from a high of 81% to a low of 76% [12]. Studies assessing uptake during the pandemic have shown increases from 2019–2020, while others examining uptake before and after the pandemic have shown decreases among certain groups, including children and healthcare personnel [10,13]. Studies conducted globally have attributed such changes in routine immunization uptake to COVID-19-related factors including decreased access to vaccines, a rise in vaccine hesitancy, and fluctuations in risk perception [14–16]. In the US, a significant shift in attitudes towards vaccines, public health, and science in general – driven largely by an increase in mis- and disinformation - has also affected vaccine uptake [17,18].

We do not fully understand the nuances of influenza vaccine uptake, or how the COVID-19 pandemic has affected uptake among different demographics. Between May 2020 and October 2024, we administered two surveys assessing self-reported influenza vaccination among US adults. Here, we present the findings of these surveys, stratified by demographics, to demonstrate the change in influenza immunization rates among US adults before and after the COVID-19 pandemic.

## Methods

### Recruitment and survey administration

Between May 2020 and October 2024, we conducted two discrete, cross-sectional national surveys assessing influenza vaccination status and intention among US adults [19,20]. Surveys were constructed using Qualtrics (Qualtrics, Provo, UT) and administered by CloudResearch, an online survey company, to a census-matched sample of US adults (18+). A representative sample was recruited from CloudResearch’s pre-existing survey panels, which consist of individuals recruited by word-of-mouth and advertising to take surveys. Respondents were recruited according to demographic quotas proportional to the U.S. Census; within each quota, respondents were selected randomly [21]. Participants were recruited from pre-existing panels of individuals enrolled in the CloudResearch survey system and received a nominal incentive (~2 USD) for completing the survey, which they could redeem in several ways, for example as cash, subscription credits, or donations. Distinct representative study populations were recruited from the same panels for all studies. Survey 1 was conducted from May 6–7, 2020, and Survey 2 from September 20–27, 2024. Sample sizes were 672 and 828 for Survey 1 and 2, respectively.

### Survey content

Survey 1 asked questions assessing respondent knowledge, attitudes, and behaviors around the COVID-19 pandemic, while Survey 2 asked questions around the 2024 mpox outbreak [19,20]. Among these questions, we asked whether respondents had received the influenza vaccine in the months prior or intended to receive it later in the season.

### Analysis

Survey results were analyzed using STATA SE 18.5. Survey weights were calculated based on estimations of age, sex, and race provided in the American Community Survey (ACS) 2022. Vaccine uptake was calculated as a combination of respondents who reported having received the vaccine and those who indicated that they were planning to receive the vaccine in the coming months. P-values less than or equal to an alpha of 0.05 were considered significant and means, proportions, and 95% confidence intervals were adjusted for survey weights.

### Ethical approvals

Approval was secured from the Institutional Review Board (IRB) at Yale University for Survey 1 (2000027891), and from the IRB at University of Texas Southwestern Medical Center for Survey 2 (STU-2024–0933). At the start of each survey, participants were advised that their results were confidential and protected, and that by proceeding with the questionnaire, they were consenting to participate. Participants were not asked to provide any identifiable data to the study team.

## Results

Over 60% of US adults surveyed in May 2020 (N = 671) indicated that they had either received the influenza vaccine recently or intended to receive it in the coming months. In contrast, 54% of US adults surveyed in October 2024 (N = 828) indicated that they had or were planning to receive the influenza vaccine.

In both 2020 and 2024, uptake was lowest among individuals aged 18–25 (47% and 50%, respectively, Table 1). At both time points, uptake was highest among individuals over the age of 55; however, this group experienced the most significant decline in uptake, with an 11% decrease between 2020 and 2024. Overall, uptake among younger individuals (<36 years old) increased, though not significantly, while uptake among older individuals (>36 years old) decreased.

**Table 1:** Change in influenza vaccination uptake from May 2020 to October 2024 (weighted).

Demographic	May 2020	October 2024	Proportion Change (95% CI)	P-value
**Age**				*0.014*
18-25	0.47	0.50	0.03 (-0.13 – 0.19)	0.698
26-35	0.55	0.58	0.03 (-0.09 – 0.15)	0.654
36-45	0.53	0.43	-0.10 (-0.22 – 0.01)	0.089
46-55	0.52	0.46	-0.05 (-0.16 – 0.06)	0.355
55+	0.74	0.62	-0.11 (-0.19 – -0.04)	**0.004**
**Gender**				*0.014*
Male	0.65	0.51	-0.13 (-0.21 – -0.06)	**0.001**
Female	0.58	0.57	-0.01 (-0.08 – 0.06)	0.726
Other	0.33	0.50	0.17 (-0.45 – 0.79)	0.598
**Race**				***0.027***
Black/African American	0.49	0.55	0.06 (-0.08 – 0.21)	0.412
American Indian/Alaska Native	0.68	0.52	-0.17 (-0.44 – 0.10)	0.235
Asian	0.64	0.54	-0.10 (-0.25 – 0.05)	0.183
Native Hawaiian/Other Pacific Islander	0.50	0.48	-0.02 (-0.75 – 0.70)	0.949
White	0.61	0.54	-0.07 (-0.13 – -0.01)	**0.030**
**Ethnicity**				*0.022*
Hispanic	0.59	0.49	-0.10 (-0.24 – 0.05)	0.196
Non-Hispanic	0.61	0.56	-0.05 (-0.11 – 0.00)	**0.050**
**Education**				*0.190*
No high school	0.20	0.22	0.02 (-0.27 – -0.32)	0.884
High school	0.54	0.50	-0.04 (-0.13 – 0.05)	0.404
Some college	0.57	0.56	-0.01 (-0.11 – -0.10)	0.896
College	0.62	0.62	0.00 (-0.09 – 0.10)	0.923
Graduate/Professional	0.71	0.55	-0.16 (-0.29 – -0.02)	**0.021**

Uptake among male respondents also decreased significantly, from 65% in 2020 to 51% in 2024 (Table 1). In 2020, fewer females than males reported being vaccinated (58%); however, uptake among females remained constant between 2020-2024, resulting in uptake among females (57%) being higher than among males in 2024.

Overall, non-White respondents were less likely to be vaccinated against influenza in 2020 than were White respondents: in 2020, only 49% of Black or African American respondents were vaccinated compared to 61% of White respondents (Table 1). However, while uptake among Black and African Americans increased slightly (+6%) from 2020 to 2024, uptake among White respondents dropped significantly, from 61% in 2020 to 54% in 2024. Similarly, while overall uptake was lower among Hispanic respondents, uptake among non-Hispanics decreased significantly (-5%) between 2020 and 2024.

In both 2020 and 2024, vaccine uptake was lowest among respondents who had not completed high school (20% and 22%, respectively, Table 1). At both timepoints, uptake rate increased with education level, except for graduate and professional degree recipients in 2024. While 71% of respondents who had completed graduate or professional-level school were vaccinated in 2020, only 55% were vaccinated in 2024, a significant decline of 16%.

## Discussion

Our results reveal an overall decrease in influenza vaccine uptake among US adults from 2020 to 2024. This decrease was notable among certain demographics: participants who were male, over 35 years of age, White, non-Hispanic, and had achieved graduate or professional degrees were significantly less likely to have been vaccinated in 2024 than they were in 2020.

The most significant decreases in uptake occurred among demographics reporting high coverage in 2020. While vaccination rates were higher among male respondents (65%) in 2020, they fell below those of female respondents in 2024, reflecting national trends [13,22,23]. Similarly, uptake among White respondents was higher than among Black and African American respondents in 2020, but fell below in 2024 [8]. While Hispanic respondents were overall less likely to be vaccinated in both 2020 and 2024, uptake among non-Hispanic respondents decreased significantly while uptake among Hispanic respondents remained constant [8]. Similarly, while less educated individuals were less likely to be vaccinated overall, individuals who had achieved the graduate and professional levels of education saw the greatest drop in uptake in 2024 [24,25]. Overall, our findings reveal significant decreases in uptake among populations traditionally associated with higher vaccine uptake. These results are in line with national estimates, which show an overall decrease in uptake, along with other studies which have shown a decline in uptake among certain groups of US adults (i.e., healthcare professionals) [10,13].

The COVID-19 pandemic has been shown to affect vaccine uptake in several ways. Globally, the pandemic interrupted the provision of routine vaccinations, and has previously been linked to lower rates of influenza vaccine uptake among demographics, including African Americans, individuals with lower socio-economic status, older adults, and healthcare workers [13]. Rates of influenza infection were lower during the pandemic, suggesting strategies employed to mitigate transmission of SARS-CoV-2 may have also reduced influenza transmission. This may have shifted Americans’ perceived need for influenza vaccination, resulting in lower intention to vaccinate despite influenza vaccines still being recommended during the pandemic [2]. Moreover, the increase in online mis- and disinformation around the COVID-19 vaccine and vaccinations in general shifted attitudes around vaccination, which may have resulted in lower uptake of the influenza vaccine [26–29].

Targeted behavioral interventions can effectively address low rates of vaccine uptake. Evidence-based strategies, which can include provider recommendation, on-site vaccination, and context-driven messaging, have been shown to improve vaccine attitudes and acceptance in several contexts, including high-income countries like the United States [30]. Importantly, healthcare professionals remain among the most reliable sources of health information in the US; as such, providers should act as trusted messengers to promote seasonal vaccinations among communities which may be unsure or unaware of the benefits of vaccination [31,32].

This messaging should not be limited to face-to-face communication: online sources of information, including the social media accounts of health professionals and authorities, gained reliability during the COVID-19 pandemic [33]. Given the importance of influenza vaccinations, and the decline in uptake seen during the pandemic, it is essential to improve uptake among US adults. The results of this survey indicate that efforts to improve uptake should include groups which have historically had higher vaccination rates. Future work should explore the impact of such targeted interventions on the demographics’ intentions and attitudes towards influenza vaccination, as well as the actual receipt of influenza vaccination.

### Limitations

This study has a few limitations. Notably, the samples were recruited from existing panels of CloudResearch survey respondents, making response bias a possibility. To mitigate this, the samples for both surveys were recruited randomly within census-matched quotas to ensure as much representation and generalizability of the US adult population as possible. We also weighted our analysis according to age, sex, and race estimations provided by ACS 2022. Additionally, these results may be affected by social desirability bias; however, participants were informed prior to taking the survey that their participation was anonymous, their results confidential, and that researchers would not know any identifying information, which should minimize social desirability bias.

## Conclusion

Our study documented an overall decrease in flu vaccine uptake in the years of the COVID-19 pandemic. US adults who are male, over 35 years of age, White, non-Hispanic, and had achieved graduate and professional-level education, reported the most significant decreases in uptake. As such, these groups may benefit from targeted behavioral interventions to improve vaccination rates.
